# A multicenter assessment of single-cell models aligned to standard measures of cell health for prediction of acute hepatotoxicity

**DOI:** 10.1007/s00204-016-1745-4

**Published:** 2016-06-25

**Authors:** Rowena L. Sison-Young, Volker M. Lauschke, Esther Johann, Eliane Alexandre, Sébastien Antherieu, Hélène Aerts, Helga H. J. Gerets, Gilles Labbe, Delphine Hoët, Martina Dorau, Christopher A. Schofield, Cerys A. Lovatt, Julie C. Holder, Simone H. Stahl, Lysiane Richert, Neil R. Kitteringham, Robert P. Jones, Mohamed Elmasry, Richard J. Weaver, Philip G. Hewitt, Magnus Ingelman-Sundberg, Chris E. Goldring, B. Kevin Park

**Affiliations:** 1 MRC Centre for Drug Safety Science, Department of Molecular and Clinical Pharmacology, University of Liverpool, Sherrington Building, Ashton Street, Liverpool, L69 3GE UK; 2 Department of Physiology and Pharmacology, Section of Pharmacogenetics, Karolinska Institutet, SE-171 77 Stockholm, Sweden; 3 Early Non-Clinical Safety, Merck KGaA, Frankfurter Str. 250, 64293 Darmstadt, Germany; 4 KaLy-Cell, 20A rue du Général Leclerc, 67115 Plobsheim, France; 5 Université de Lille 2, EA 4483, Lille, France; 6 Biologie Servier, 905 Rue de Saran, 45520 Gidy, France; 7 UCB BioPharma SPRL, Non-Clinical Development, Chemin du Foriest, 1420 Braine-l’Alleud, Belgium; 8 Sanofi-Aventis Recherche and Développement, Drug Safety Evaluation, Alfortville, France; 9 Sanofi-Aventis Deutschland GmbH, R&D DSAR, Preclinical Safety FF, Industriepark Hoechst, Building H823, Room 104, 65926 Frankfurt am Main, Germany; 10 GSK, Medicines Research Centre, Gunnels Wood Road, Stevenage, Hertfordshire SG1 2NY UK; 11 GSK, David Jack Centre for R&D, Park Road, Ware, Hertfordshire SG12 0DP UK; 12 AstraZeneca, Innovative Medicines and Early Development, Drug Safety and Metabolism, ADME Transporters, Unit 310 – Darwin Building, Cambridge Science Park, Milton Road, Cambridge, CB4 0FZ UK; 13 North Western Hepatobiliary Unit, Aintree University Hospital NHS Foundation Trust, Longmoor Lane, Liverpool, L9 7AL UK; 14 Université de Franche-Comté, EA 4267, 25030 Besançon, France

**Keywords:** Pharmaceuticals, Cytotoxicity, Hepatocytes, Predictive toxicology, Toxicity, Acute

## Abstract

Assessing the potential of a new drug to cause drug-induced liver injury (DILI) is a challenge for the pharmaceutical industry. We therefore determined whether cell models currently used in safety assessment (HepG2, HepaRG, Upcyte and primary human hepatocytes in conjunction with basic but commonly used endpoints) are actually able to distinguish between novel chemical entities (NCEs) with respect to their potential to cause DILI. A panel of thirteen compounds (nine DILI implicated and four non-DILI implicated in man) were selected for our study, which was conducted, for the first time, across multiple laboratories. None of the cell models could distinguish faithfully between DILI and non-DILI compounds. Only when nominal in vitro concentrations were adjusted for in vivo exposure levels were primary human hepatocytes (PHH) found to be the most accurate cell model, closely followed by HepG2. From a practical perspective, this study revealed significant inter-laboratory variation in the response of PHH, HepG2 and Upcyte cells, but not HepaRG cells. This variation was also observed to be compound dependent. Interestingly, differences between donors (hepatocytes), clones (HepG2) and the effect of cryopreservation (HepaRG and hepatocytes) were less important than differences between the cell models per se. In summary, these results demonstrate that basic cell health endpoints will not predict hepatotoxic risk in simple hepatic cells in the absence of pharmacokinetic data and that a multicenter assessment of more sophisticated signals of molecular initiating events is required to determine whether these cells can be incorporated in early safety assessment.

**Electronic supplementary material:**

The online version of this article (doi:10.1007/s00204-016-1745-4) contains supplementary material, which is available to authorized users.

## Introduction

Drug-induced liver injury (DILI) poses a serious issue not only for patients and healthcare professionals, but also for the pharmaceutical industry and regulatory authorities. This is mainly due to the occurrence of human-specific and idiosyncratic adverse reactions at the clinical and post-marketing stages, thus leading to the termination of drug development, black box warnings or even withdrawal of drugs from the market (Bell and Chalasani 2009; Marino et al. 2001).

In vitro models, such as the human hepatocarcinoma cell line HepG2, are widely used across the pharmaceutical and chemical industries, as an initial screen to determine the likely risk of new chemical entities (NCEs) eliciting DILI in man. This is because (1) they can be applied quickly to generate reproducible data, (2) they are amenable to high-content screening and (3) they are relatively cheap and readily available (Gerets et al. 2012; O’Brien et al. 2006). Although not of human origin, rodent liver-derived cell lines and primary hepatocytes (which are more readily available compared with their human counterpart) are also routinely used. However, interspecies differences are known to have a significant impact on predictivity of DILI and non-DILI compounds (Olson et al. 2000). On the other hand, primary human hepatocytes (PHH) are not generally used in such a first screen as they exhibit inter-individual variation, thereby potentially confounding analysis and interpretation of intra-study and/or intra-project results between different NCEs; they are also expensive to acquire, and they undergo significant de-differentiation during culture (Guillouzo et al. 2007; Madan et al. 2003; Richert et al. 2006). However, PHH cultured in vitro are still considered to be useful in the testing paradigm, as the nearest representation of the key metabolically active cell of the liver.

Since DILI continues to pose significant problems in drug development, this suggests that currently used in vitro models are not appropriate for effective screening, but a comprehensive, multicenter, unbiased assessment to test this unequivocally has never been performed. Therefore, the Innovative Medicines Initiative (IMI)-funded consortium ‘Mechanism-based Integrated Systems for the Prediction of Drug-Induced Liver Injury’ (MIP-DILI) has assessed current in vitro cell models, using an evidence-based panel of compounds implicated in DILI in man, in order to determine whether any of these simple cell models *per se* are actually predictive of human DILI. Furthermore, by using a small panel of DILI- and non-DILI-implicated compounds, and basic measures of cell health, we were able to monitor reproducibility across different sites, thereby ensuring that our data should be more definitive than any currently available.

## Materials and methods

**Fig. 1:**
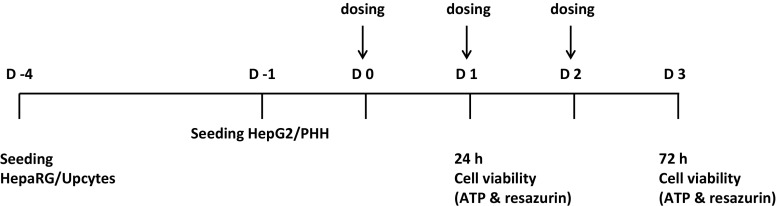
Cytotoxicity study design. Primary human hepatocytes (cryopreserved and fresh), HepG2 (ECACC and TS clones), HepaRG (cryopreserved and fresh), and Upcyte cells were seeded on the days indicated followed by exposure to the thirteen training compounds (Table 4) as detailed in the method section for 24 or 72 h. After compound treatment, cell viability was assessed by measurement of intracellular ATP and resorufin which is the product when resazurin is reduced (a measure of cellular metabolism)

PHH, HepG2, HepaRG and Upcyte cells (Table 1) in conjunction with a particular endpoint (referred to as cell models) were evaluated using harmonized protocols which were designed and agreed by all test site participants as detailed below, for their ability to predict DILI liability of NCEs. For the assessment of each cell model, the same protocol was used by all the test sites involved and the supplier and product codes of all materials and reagents included. Each training compound was sourced by all test sites from the same supplier and acquired the same batch/lot number. Plasticware and other reagents such as media and media supplements were sourced by all the test sites from the suppliers and product codes stated in the protocols; however, standardization did not extend to sourcing these items from specific batch/lot numbers. To determine inter-laboratory variation, several of the cell models (cryopreserved PHH, cryopreserved HepaRG, HepG2/ECACC and Upcyte cells) were evaluated by at least two test sites (Table 2). Furthermore, a simple experimental protocol was designed (Fig. 1) and basic endpoints were chosen for our cell models, i.e., resorufin and ATP assays for the assessment of cell viability, to allow generation of rapid data by the test sites involved that could be easily compared.

**Table 1:** Cell types assessed for their ability to predict DILI

Cell type	Description	Uses in DILI and hepatotoxicity screening	References
HepG2	Human hepatocellular carcinoma cell line, limited metabolic capacity compared with PHH (clone-dependent)	Commonly used for early cytotoxicity screening	(Gerets et al. 2012; Hewitt and Hewitt 2004)
HepaRG	Human hepatocarcinoma cell line, co-culture of hepatocytes and cholangiocyte-like cells, metabolic capacity comparable with PHH without donor variation	Potential use in in vitro drug metabolism studies	(Antherieu et al. 2010; Gripon et al. 2002; Lambert et al. 2009)
Primary human hepatocytes	Isolated human hepatocytes, gold standard in in vitro testing. Limitations in availability and inter-individual variability	Considered to be the gold standard for in vitro drug metabolism and cytotoxicity testing	(Guillouzo et al. 2007; Madan et al. 2003)
Upcyte hepatocytes	Transduced human hepatocytes with genes that upregulate proliferation. Basal expression of metabolizing enzymes comparable with 5 day culture of PHH	New cell model, advantage of ‘unlimited’ number of cells from one donor	(Burkard et al. 2012)

**Table 2:** Participants in the multicenter assessment of the seven cell models

Test site	Cell models						
	Primary human hepatocytes		HepaRG		HepG2		Upcyte
	Fresh	Cryopreserved	Fresh	Cryopreserved	ECACC	TS	
GSK (Hertfordshire, UK)		●			●	●	
KaLy-Cell (Plobsheim, France)		●					
Merck (Darmstadt, Germany)				●	●		●
Liverpool University (Liverpool, UK)	●	●			●		
Sanofi Aventis (Alfortville and Frankfurt am Main, Germany)				●	●		
Servier (Gidy, France)			●				●
UCB (Braine-l’Alleud, Belgium)							●

### Cell culture

The different seeding densities used are the optimal for each cell type. Data generated from each cell model was first analyzed relative to the corresponding untreated controls and then comparisons were made across the different cell models. Donor details for the fresh and cryopreserved PHH as well as the Upcyte cells are summarized in Table 3. Allocation of PHH donors for each test site is also summarized in Table 3. Pre-qualification for choosing the five cryopreserved PHH donors was based on their viability post-thawing, ability to adhere to cell culture plates, metabolic profile and material availability to ensure there was sufficient supply for the planned experimental work and any future studies. Donor demographics were collected as summarized in Table 3; however, these were not considered a priority during the pre-qualification stage as the aim was to select a cohort of the population in an unbiased manner.

**Table 3:** Cryopreserved and fresh PHH and Upcyte cells donor information

ID	Gender	Age	Ethnicity	Pathology	Medication
**Primary human hepatocytes**					
Cryopreserved					
B17042008*	♀	69	Caucasian	Breast cancer	Not available
M1603LT^#^	♀	68	Caucasian	Colorectal adenocarcinoma	Tardyferon, Lercan, Previscan, Contramal, Cétornan
S1100T^#^	♂	59	Caucasian	Colorectal adenocarcinoma	None
S1070T^§^	♂	69	Caucasian	Not available	Not available
S1099T^§^	♀	56	Caucasian	Colorectal adenocarcinoma	Levothyrox, Amlodipine, DiffuK, Ogast
Fresh					
UoL49	♂	66	Caucasian	Not available	Not available
**Upcyte cells**					
422A	♂	0	Hispanic	Anoxia	None

* Tested by all three test sites involved in assessing cryopreserved PHH (GSK, KaLy-Cell, and UoL, please refer to Table 2)

^#^Tested by KaLy-Cell

^§^Tested by UoL

## Cryopreserved primary human hepatocytes

Cryopreserved PHH from five donors were provided by KaLy-Cell (Plobsheim, France). The human biological samples were sourced ethically and their research use was in accord with the terms of the informed consents. The hepatocytes were thawed in KLC-Thawing Medium (KLC-TM; proprietary medium composition), centrifuged (168×*g*; 20 min; room temperature), washed in KLC-Washing Medium (KLC-WM; proprietary medium composition; 100×*g*; 5 min; room temperature) and resuspended in KLC-Seeding Medium (KLC-SM; Williams’ Medium E (Life Technologies, Paisley, UK) supplemented with 10 % heat-inactivated fetal calf serum (FCS, Life Technologies, Paisley, UK), 1 µM dexamethasone (Sigma-Aldrich, St. Louis, MO, USA), 4 µg/mL insulin [(Life Technologies, Paisley, UK) and 10 units penicillin/10 µg streptomycin (Life Technologies, Paisley, UK)]. Cell number and viability were determined by the trypan blue exclusion method, and the cells were plated at a seeding density of 70,000 cells/well of a KaLy-Cell home-coated type I rat tail collagen (10 µg/well) 96-well plate. This seeding density has been optimized and recommended by KaLy-Cell to achieve ≥80 % confluence in a 96-well plate format 24 h after plating. The cells were allowed to attach for 4–6 h (37 °C; 5 % CO_2_; 95 % air) after which the cells were overlaid with 0.25 mg/mL matrigel in KLC-Seeding Medium and left to incubate overnight (37 °C; 5 % CO_2_; 95 % air). Cells were used for analysis if the attachment efficiency was ≥80 %. Serum-free KLC-Maintenance Medium (KLC-MM; Hepatocyte Maintenance Medium (Lonza, Basel, Switzerland) supplemented with 1 µM dexamethasone (Sigma-Aldrich, St. Louis, MO, USA), 4 µg/mL insulin (Life Technologies, Paisley, UK) and 10 units penicillin/10 µg streptomycin (Life Technologies, Paisley, UK) was used for compound treatment.

## Fresh primary human hepatocytes

Fresh primary human hepatocytes isolated from a different donor (UoL49) to the five cryopreserved PHH donors were also included in our analysis. A liver resection from donor UoL49 was received as surgical waste from Aintree Hospital, Liverpool, United Kingdom, with full patient consent and ethical approval from the relevant authorities (National Research Ethics Service REC reference: 11/NW/0327). Upon collection, the specimen was immediately transported to the University of Liverpool, Liverpool, United Kingdom, on ice in HEPES-buffered saline [HBS; 10 mM Hepes (Sigma-Aldrich, St. Louis, MO, USA), 5 mM KCl (Fisher Scientific, Loughborough, UK), 136 mM NaCl (Fisher Scientific, Loughborough, UK) and 0.5 % glucose (Fisher Scientific, Loughborough, UK)]. Hepatocyte isolation was performed using a 2-step collagenase method as described previously (LeCluyse et al. 2005). Briefly, blood was removed from the liver tissue by blanching through perfusion with HBS pre-warmed to 37 °C and subsequently digested with 0.25 mg/mL collagenase A/IV (Sigma-Aldrich, St. Louis, MO, USA) in HBS containing 0.7 mM CaCl_2_ (Fisher Scientific, Loughborough, UK), also pre-warmed to 37 °C. The capsule was opened and the hepatocytes released and collected in cold Williams’ Medium E (Sigma-Aldrich, St. Louis, MO, USA). The hepatocytes were separated from the undigested tissue remnants with a 125-μm mesh, washed twice with cold Williams’ Medium E by centrifugation (80×*g*; 5 min; 4 °C), and the hepatocytes resuspended in cold complete Williams’ Medium E [supplemented with 1 % insulin–transferrin–selenium (Life Technologies, Paisley, UK), 2 mM l-glutamine (Sigma-Aldrich, St. Louis, MO, USA), 100 nM dexamethasone (Sigma-Aldrich, St. Louis, MO, USA), and 1 % penicillin/streptomycin (Sigma-Aldrich, St. Louis, MO, USA)]. Cell number and viability were determined using the trypan blue exclusion method, and the cells were plated at a seeding density of 100,000 cells/well in a BD Biocoat Collagen I 96-well plate (Becton and Dickinson, New Jersey, USA). This seeding density was determined to be optimal for these cells in a 96-well plate format to achieve ≥80 % confluence 24 h after plating. The cells were allowed to attach for 3 h (37 °C; 5 % CO_2_; 95 % air), the incubation medium removed, and the attached cells overlaid with 0.25 mg/mL matrigel (Becton and Dickinson, New Jersey, USA) in cold complete Williams’ Medium E. The cells were further incubated overnight (37 °C; 5 % CO_2_; 95 % air) prior to dosing. The cell viability after hepatocyte isolation was 97 %, and the cells were 85 % confluent 16 h post-plating. Complete Williams’ Medium E was serum-free and was also used for compound treatment.

## Cryopreserved HepaRG cells

Differentiated cryopreserved HepaRG cells were purchased from Biopredic International (Saint-Gregoire, France). The cells were thawed in Thawing/Plating Medium [Williams’ Medium E supplemented with 1 % penicillin/streptomycin, 1 % l-Glutamine, 10 % FCS and 12.5 % ADD670 cocktail (BioPredic International, Saint-Gregoire, France; proprietary medium composition)], centrifuged (500×*g*; 5 min; room temperature) and then resuspended in fresh Thawing/Plating Medium. After the addition of the ADD670 cocktail, the final DMSO concentration in the Thawing/Plating Medium was 0.5 %. Cell number and viability were determined by the trypan blue exclusion method, and the cells were plated at a seeding density of 72,000 cells/well in white/clear bottom 96-well plates (Beckton Dickinson, New Jersey, USA). The cells were cultured (37 °C; 5 % CO_2_; 95 % air) for 24 h after which the Thawing/Plating Medium was replaced with a specified Tox medium (Williams’ Medium E supplemented with 1 % penicillin/streptomycin, 1 % l-Glutamine, and 12.5 % ADD650 cocktail [Biopredic International, Saint-Gregoire, France, proprietary medium composition]). The Tox medium was serum- and DMSO-free. Cells were incubated for another 48 h without additional medium change and subsequently used for experimentation, if the attachment efficiency was at or above 85 %. For compound treatment, the Tox medium was used.

## Fresh HepaRG cells

Undifferentiated HepaRG cells were purchased from Biopredic International (Saint-Gregoire, France). For differentiation, one vial of cells was thawed and seeded in a 75-cm^2^ flask in proliferation medium [Williams’ Medium E supplemented with 2 mM l-Glutamine, 10 % fetal calf serum and ADD710 cocktail (BioPredic International, Saint-Gregoire, France; proprietary medium composition)]. The proliferation medium did not contain DMSO. The cells were allowed to recover and proliferate in culture (37 °C; 5 % CO_2_; 95 % air) for 2 weeks with a medium change of every 2–3 days. The proliferation medium was then replaced with the differentiation medium [Williams’ Medium E supplemented with 2 mM l-Glutamine, 10 % fetal calf serum, and ADD720 cocktail (Biopredic International, Saint-Gregoire, France; proprietary medium composition)]. After the addition of the ADD720 cocktail, the final DMSO concentration in the differentiation medium was 1.7 %. The cells were maintained in culture for 2 weeks (37 °C; 5 % CO_2_; 95 % air) after which the cells have reached a hepatocyte-like morphology. Differentiated cells were harvested and seeded in 96-well plates at a density of 72,000 cells/well in differentiation medium. The cells were used for the toxicity study 5 days after seeding. For compound treatment, serum- and DMSO-free Tox medium was used [Williams’ Medium E supplemented with 2 mM l-Glutamine and ADD650 cocktail (Biopredic International, Saint-Gregoire, France; proprietary medium composition)].

## HepG2/ECACC and HepG2/TS cells

A specific clone of HepG2 cells was purchased from the European Collection of Cell Cultures (ECACC). The cells were cultivated and low passage frozen stocks were generated and banked by one MIP-DILI partner designated as the cell bank. The cells were distributed to the relevant MIP-DILI partners. The HepG2 cells were cultured in Dulbecco’s Modified Eagle Medium (DMEM; Lonza, Basel, Switzerland) supplemented with 10 % FBS (Lonza, Basel, Switzerland), 1 % penicillin/streptomycin (Lonza, Basel, Switzerland), 1 % l-glutamine (Lonza, Basel, Switzerland) and 1 % non-essential amino acids (NEAA; Sigma-Aldrich, St. Louis, MO, USA) and passaged upon reaching 80 % confluence using Trypsin–EDTA (Lonza, Basel, Switzerland). Cell number and viability were determined using the trypan blue exclusion method, and the cells were plated at a seeding density of 20,000 cells/well in BD Biocoat Collagen I 96-well plates (Beckton Dickinson, New Jersey, USA). The cells were then incubated for 24 h (37 °C; 5 % CO_2_; 95 % air) prior to compound treatment during which serum-free culture medium was used. To determine whether there are differences in response between two clones of the same cell type, an in-house clone of HepG2 from one test site referred to as the HepG2/TS clone was also assessed for its sensitivity to the compounds and compared with the HepG2/ECACC clone. The same conditions were applied during the culture of the HepG2/TS cells with the exception of using a NucleoCounter (NC-100, Chemometec) for cell counting, TrypLE (Life Technologies, Paisley, UK) for passaging, and the seeding density being 30,000 cells/well.

## Upcyte hepatocytes

Preliminary assessment of two Upcyte hepatocyte donors with regards to their sensitivity to a small set of compounds was performed prior to the current study and showed no significant difference (data not presented). Furthermore, despite the infant age of the donor (422A) used in the current study, the basal metabolic profile of this donor including phase I and II enzyme activities which are crucial in the clearance of compounds from the body, as provided by the supplier, was not dissimilar to the adult donors. As such, the use of donor 422A was ultimately due to the large amount of material available to allow for future studies to be carried out should the need arise.

Upcyte hepatocytes from donor 422A (Medicyte, Heidelberg, Germany) were thawed in Upcyte Thawing Medium [Upcyte High Performance Medium (Medicyte, Heidelberg, Germany) and 10 % FCS (Fisher Scientific, Paisley, UK)]. The cells were then centrifuged (90×*g*; 5 min; room temperature) and resuspended in fresh Upcyte High Performance Medium (Medicyte, Heidelberg, Germany). Cell number and viability were determined via the trypan blue exclusion method, and the cells were seeded at 18,750 cells/well in white/clear bottom 96-well plates (Beckton Dickinson, New Jersey, USA). After seeding, the cells were incubated for 72 h (37 °C; 5 % CO_2_; 95 % air) without additional medium change prior to compound treatment.

### Dosing

For this study, DILI compounds are defined as those that have been reported to cause hepatotoxicity in man while non-DILI compounds have not. Postulated mechanisms of hepatotoxicity of the DILI-implicated compounds and corresponding references can be found in Table 4. Final concentrations used in the cell incubations for all compounds are also detailed in Table 4. The compound concentrations used were chosen as representative for each compound when conducting an in vitro safety assessment study. All stock solutions, except for metformin, were prepared as 200-fold stocks in DMSO (Sigma-Aldrich, St. Louis, MO, USA). Stock aliquots were stored at −20 °C and only thawed once. Metformin stock solutions were prepared as 200-fold stocks in distilled water. Cells were dosed either once and exposed for 24 h or once daily over a 3-day period for a 72 h exposure. For the repeat dose, the old dosing solution was discarded each day and replaced with fresh dosing solution to ensure that the compound and DMSO concentrations in which the cells were exposed to were constant throughout the 72-h period. Dosing solutions were prepared fresh every day. To minimize or prevent non-specific binding to proteins, all media used for dosing did not contain serum. Cells were dosed in technical triplicates with a final concentration of 0.5 % DMSO. Controls were cells treated with 0.5 % DMSO in dosing medium, in the absence of compounds.

**Table 4:** The panel of compounds used in this study (nine implicated in risk of DILI; four without known DILI liability), including concentrations used, and putative mechanism/s of hepatotoxicity in man

Compound	Hepatotoxic/non-hepatotoxic	Final dose concentrations (μM)	Therapeutic function(s)	Postulated toxic mechanisms	References
Amiodarone	Hepatotoxic	3, 5, 10, 30, 50, 100, 300	Antiarrythmic	b, d	(Bandyopadhyay et al. 1990; Dake et al. 1985; Pourbaix et al. 1985)
Bosentan	Hepatotoxic	3, 5, 10, 30, 50, 100, 300	Antihypertensic	c	(Fattinger et al. 2001; Gutierrez et al. 2013)
Buspirone	Non-hepatotoxic	3, 5, 10, 30, 50, 100, 300	Anxiolytic	Non-hepatotoxic	(Sakr and Andheria 2001; Zhu et al. 2005)
Diclofenac	Hepatotoxic	10, 30, 50, 100, 300, 500, 1000	Analgesic	a, b, c	(Tujios and Fontana 2011)
Entacapone	Non-hepatotoxic	10, 30, 50, 100, 300, 500, 1000	Parkinson’s disease	Non-hepatotoxic	(Heikkinen et al. 2001; Lautala et al. 2000)
Metformin	Non-hepatotoxic	30, 50, 100, 300, 500, 1000, 3000	Antidiabetic	Non-hepatotoxic	(Tucker et al. 1981; Tzvetkov et al. 2009)
Nefazodone	Hepatotoxic	3, 5, 10, 30, 50, 100, 300	Antidepressant	a, c	(Barbhaiya et al. 1996; Kalgutkar et al. 2005; Kostrubsky et al. 2006)
Paracetamol	Hepatotoxic	30, 100, 300, 1000, 3000, 10,000, 30,000	Analgesic	a	(Dahlin et al. 1984; Sevilla-Tirado et al. 2003)
Perhexiline	Hepatotoxic	3, 5, 10, 30, 50, 100, 300	Antianginal	c, d	(Amoah et al. 1986; Fromenty and Pessayre 1997)
Pioglitazone	Non-hepatotoxic	3, 5, 10, 30, 50, 100, 300	Antidiabetic	Non-hepatotoxic	(Rajagopalan et al. 2005; Wong et al. 2004)
Tolcapone	Hepatotoxic	10, 30, 50, 100, 300, 500, 1000	Parkinson’s disease	a, c	(Jorga et al. 1999; Lautala et al. 2000; Smith et al. 2003)
Troglitazone	Hepatotoxic	3, 5, 10, 30, 50, 100, 300	Antidiabetic, anti-inflammatory	a, b, c, d	(Kaplowitz 2005; Loi et al. 1999; Loi et al. 1997)
Ximelagatran	Hepatotoxic	3, 5, 10, 30, 50, 100, 300	Anticoagulant	e	(Keisu and Andersson 2010; Schutzer et al. 2004)

Key *a* reactive metabolites, *b* mitochondrial dysfunction, *c* BSEP inhibition, *d* lysosomal dysfunction, *e* immune-mediated

### Resorufin assay

After incubation with the compounds, cell viability was determined using a 4.5 mM stock solution of resazurin (Sigma-Aldrich, St. Louis, MO, USA) prepared in phosphate buffer. This stock solution was added into the cells at 10 % of the cell culture volume to give a final concentration of 450 µM resazurin. The cells were incubated for 1 h (37 °C; 5 % CO_2_; 95 % air) after which the medium samples were transferred into black, flat-bottomed 96-well plates. Reduction of the resazurin dye results in the highly fluorescent product, resorufin which is measured at 530–560 nm excitation wavelength and 590 nm emission wavelength.

### ATP assay

The CellTiter Glo assay kit (Promega, Madison, WI, USA) was used to determine the ATP content in the cells after exposure to the thirteen compounds for 24 or 72 h. After incubation with the compounds, the cells were washed twice with PBS and added with fresh PBS and CellTiter Glo solution in equal volumes. The cells were placed in a plate shaker for 2 min to induce lysis and left to incubate for 10 min at room temperature. The supernatant samples were transferred into opaque flat-bottomed 96-well plates (Greiner-Bio-One, Frickenhausen, Germany) and the luminescence measured.

### Data analysis

Cell viability was determined as the percentage of the fluorescent resorufin after incubation with resazurin in the treated cells compared with vehicle control or the percentage of ATP detected in the treated cells compared with vehicle control. Individual EC_50_ values from all participants were calculated using GraphPad Prism 6 (version 6.03) at one partner site. Concentration series for each compound and cell type were log-transformed and fitted with a sigmoidal regression function assuming a lower viability plateau with increasing compound concentration of 0 and a Hill coefficient of −1 using Python and Prism (GraphPad Software). Differences between two dilution series were computed using F-tests. Differences were considered significant when *p* < 0.05 and, to compensate for biological significance, when relative EC_50_-differences > three-fold. ‘No fit’ indicates no decrease in viability of at least one compound in the respective pairwise comparison. Variability coefficients were calculated by dividing the number of tests with significantly different responses by the number of all pairwise comparisons. Hierarchical clustering was performed using Qlucore Omics Explorer 3.1 on mean-centered sigma-normalized data using maximum linkage. Correlation of ATP and resorufin values was calculated using Pearson correlation. Statistical difference between 24 and 72 h toxicity measurements was computed using a paired Student’s *t* test. In order to relate compound concentrations leading to loss of viability to therapeutic concentrations, the EC_50_ values were divided by the corresponding C_max_ values (EC_50_/C_max_). References used to obtain these values (µM) are provided in Table 4: amiodarone—0.807; nefazodone—0.859; paracetamol—139; tolcapone—27.81; diclofenac—7.44; ximelagatran—0.3; troglitazone—6.387; perhexiline—1.525; bosentan—7.4; buspirone—0.005; entacapone—1.5; metformin—7.74; pioglitazone—2.672.

## Results

The primary aim of this study was to determine, in an unbiased fashion, if several simple cell models (defined as a cell type in conjunction with a specific endpoint), currently used in industry, can distinguish between NCEs with respect to their potential to cause DILI in man, in the absence of exposure data. Nine compounds that are associated with human DILI and four that are not (Table 4) were investigated using four simple cell types: PHH, HepG2, HepaRG and Upcyte cells. In order for this study to be conducted in a standardized manner across multiple centers in Europe (for study scheme and participants, see Fig. 1 and Table 2, respectively), two simple endpoints, ATP and resorufin, were measured to assess toxicity. Our results demonstrated good correlation between these two endpoints for all compounds, time points and cells (*r* = 0.94, Supplementary Table 1); therefore, only ATP data are shown in the figures in the main body of the paper, for the sake of clarity.

**Fig. 2:**
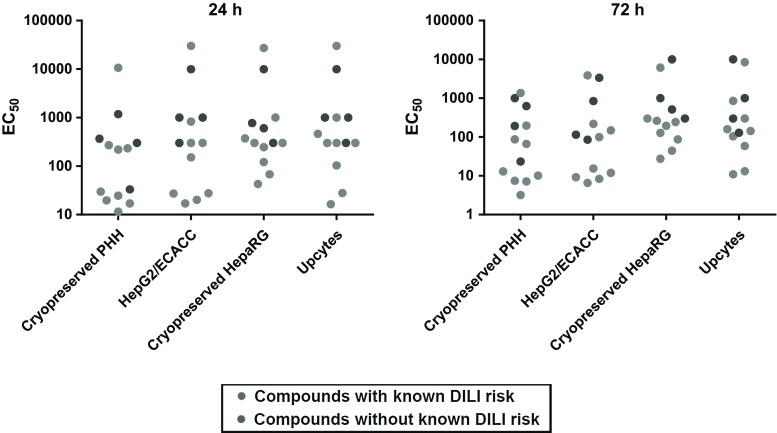
Simple-cell models using a basic measure of cell health cannot discriminate between DILI-implicated and non-DILI-implicated compounds. Scattergram of EC_50s_ derived from intracellular ATP content measurements after exposure to the training compounds of each cell type for 24 (**a**) or 72 h (**b**), expressed as the mean of multiple determinations carried out across all the test sites involved in assessing each cell type

To assess the primary aim of our study, the EC_50_ values for the ATP response to each of the compounds grouped as DILI and non-DILI, by the HepG2/ECACC, cryopreserved PHH, cryopreserved HepaRG and Upcyte cells after a 24 h and 72 h exposures, are shown in Fig. 2. No clear segregation between the DILI and non-DILI compounds was observed based on the EC_50_ values for each compound per cell type demonstrating that none of the cell models were able to faithfully distinguish between DILI and non-DILI compounds, at either time point.

**Fig. 3:**
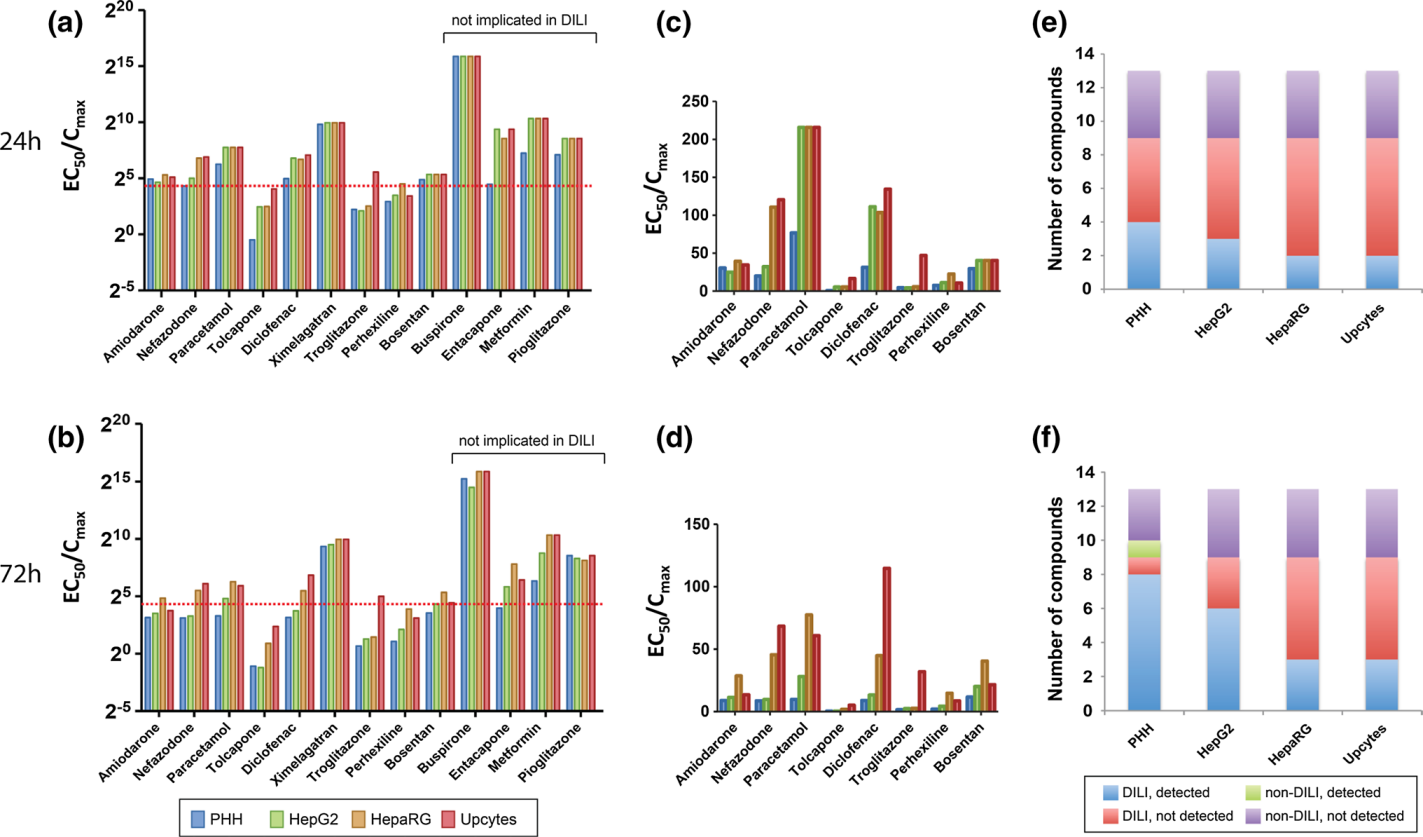
When C_max_ data are available, primary human hepatocytes are the most sensitive of the cell models for the assessment of cellular toxicity. **a**, **b** Clustered column plots showing the EC_50_/C_max_ values for all thirteen compounds as detected by all seven forms of the cell models assessed after 24 (**a**) and 72 h (**b**). The four compounds not implicated in DILI are indicated. **c**, **d** DILI-implicated compounds after 24- (**c**) and 72-h exposure (**d**) are shown on a linear axis to aid comparisons between the different cell models. **e**, **f** Stacked column plots visualizing the number of compounds classified as toxic by the different cell models with a EC_50_/C_max_ of 20 (*red line* in panel a) after 24 (**e**) and 72 h (**f**). Note that primary human hepatocytes (PHH) are the most sensitive cell type recognizing 8 of the 9 compounds implicated in DILI after 72 h (color figure online)

We then corrected for in vivo exposure by calculating EC_50_ (ATP)/C_max_ for each compound in each cell model at 24 and 72 h (Fig. 3a–f). For clarity, Fig. 3c and d are on a linear scale to aid comparisons between the cell models. Importantly, while no cell model was able to distinguish between DILI- and non-DILI-implicated compounds based on primary EC_50_ values as obtained in the early stages of drug development, primary human hepatocytes and, to a lesser extent, HepG2 cells can indicate DILI risks when exposure levels are accounted for. As no unequivocal critical EC_50_/C_max_ is defined in the literature, we assessed how many DILI compounds were classified as potentially hepatotoxic using a range of values (Supplementary Fig. 1). With an EC_50_/C_max_ of 20 (Fig. 3e, f), all DILI-implicated compounds are recognized as such in PHH after 72 h (but not after 24 h), with the exception of ximelagatran, while only one of the control compounds falls below the EC_50_/C_max_ = 20 level (entacapone). With the exception of paracetamol, HepG2 cells generated similar profiles to the cryopreserved PHH after exposure to the compounds for 72 h (Fig. 3d).

**Fig. 4:**
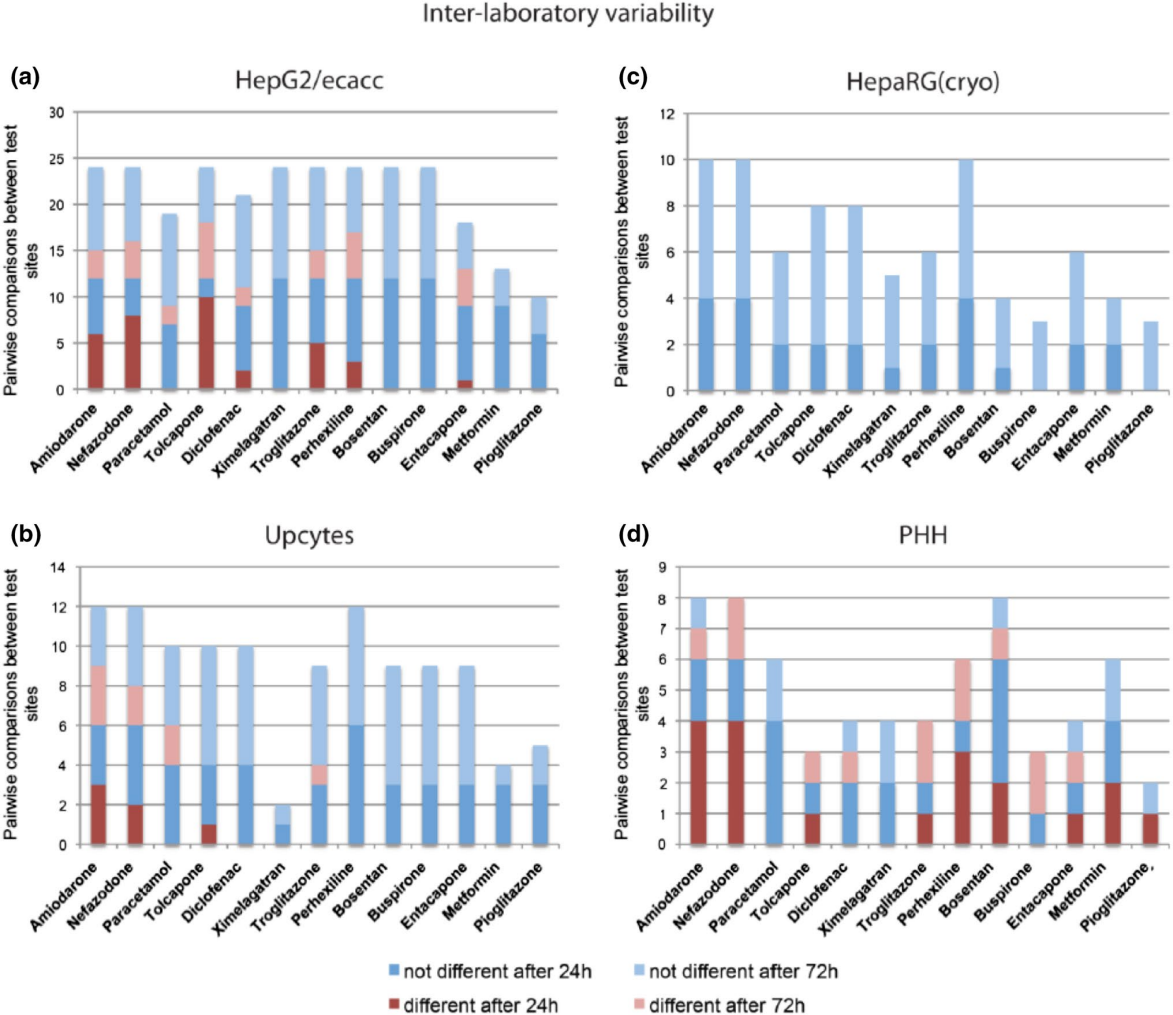
Degree of inter-laboratory variability depends on the cell model and the compound. *Stacked column plots* demonstrating the degree of inter-laboratory variability for HepG2/ecacc (**a**), Upcyte (**b**), cryopreserved HepaRG cells (**c**), and cryopreserved PHH (**d**). On the y-axis, the number of different (*shades of red*) and not different (*shades of blue*) pairwise comparisons between the participating partner laboratories is depicted. While HepaRG cells and Upcytes (12.3 %) show no or minor variability between laboratories, HepG2/ecacc (23.4 %) and primary human hepatocytes (48.5 %) elicit variable responses (color figure online)

We then explored inter- and intra-laboratory variation in our data, the effect of inter-donor and clonal variation, as well as differences in sensitivity to the compounds between fresh and cryopreserved cells. As part of our experimental design, cryopreserved PHH, HepG2/ECACC, cryopreserved HepaRG, and Upcyte cells were evaluated by at least two test sites to allow pairwise comparison of the cytotoxicity profiles generated (Table 2). For each of the cell models evaluated, the statistical significance of all pairwise comparisons between cytotoxicity curves was determined. We detected statistically significant inter-laboratory variation in the cytotoxicity profiles generated by each test site that assessed cryopreserved PHH, HepG2/ECACC, and Upcyte cells for their response to all the training compounds. These differences appeared to be of varying degrees, while the Upcytes (12.3 %) showed minor variability between the laboratories, and the HepG2/ECACC (23.4 %) and primary human hepatocytes (48.5 %) elicited clear variable responses. On the other hand, the HepaRG cells showed no difference in all pairwise comparisons performed. Furthermore, the degree in which each compound varied per cell type across the relevant test sites also differed with the exception of ximelagatran, for which no toxicity was detected even at the highest doses despite the use of harmonized protocols (Fig. 4). As such, the inter-laboratory differences appeared to be cell type- as well as compound-dependent.

Similar to our findings on inter-laboratory variability, differing degrees of intra-laboratory variability were observed between cell types. In particular, HepG2 (15.4 %) and Upcyte (11.5 %) cells differed markedly between replicate experiments in the same laboratory while HepaRG (5.7 %) and PHH (2 %) varied considerably less. In addition, intra-laboratory analysis was also observed to vary between compounds ranging from no significant variation between replicate experiments observed with paracetamol and diclofenac compared with pioglitazone demonstrating differences in 21.1 % of comparisons. Based on these results, we conclude that intra-laboratory variation and thus result reproducibility can also differ between cell types and compounds.

**Fig. 5:**
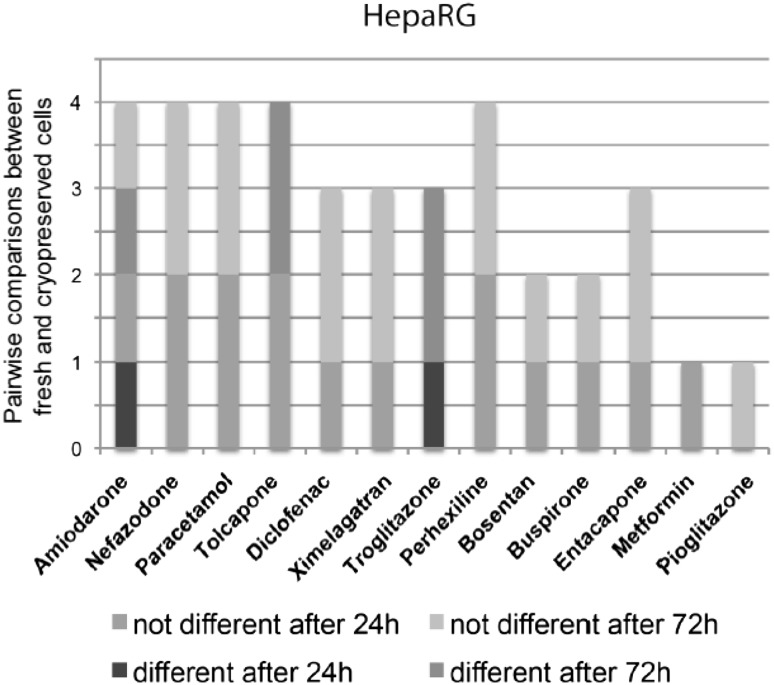
Fresh and cryopreserved cells elicit overall similar responses to the compounds. *Stacked column plots* showing the responses of fresh and cryopreserved HepaRG cells. On the y-axis, the number of different (*shades of red*) and not different (*shades of blue*) pairwise comparisons between fresh and cryopreserved cells are plotted. Differences in responses are detected in 18.4 % (HepaRG) of all pairwise comparisons (color figure online)

In order to examine differences in compound sensitivity between cryopreserved and fresh cells, cryopreserved and fresh HepaRG cells were compared in their response to the compounds tested (Fig. 5). We observed a biologically significant difference between cryopreserved and fresh HepaRG cells for only three compounds. Similarly, we also compared the cytotoxicity profiles generated for each of the training compounds from one fresh PHH donor with the five cryopreserved donors and found a biologically significant difference for two compounds (Supplementary Fig. 2).

**Fig. 6:**
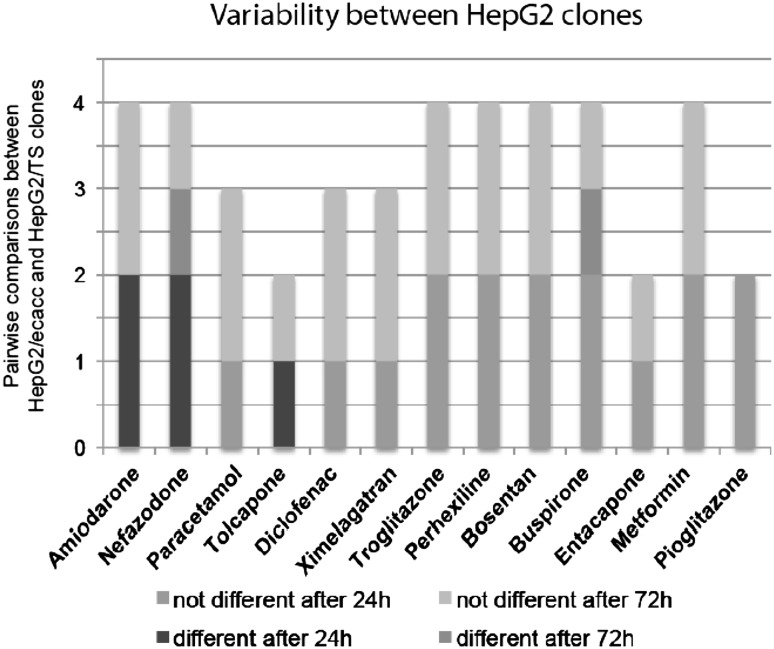
Comparison of the responses of two commonly used HepG2 clones indicates only minor differences between clones. *Stacked column plot* showing the differences in response to the compounds between HepG2/ecacc and HepG2/TS clones. On the y-axis, the number of different (*shades of red*) and non-different (*shades of blue*) pairwise comparisons is depicted. While responses to amiodarone and nefazodone are dissimilar, only few differences in the response to the other compounds are detected (overall 16.2 % differences) (color figure online)

Differences in basal activities of phases I and II enzymes have been reported in HepG2 cells that have been obtained from different sources (Hewitt and Hewitt 2004). Therefore, we determined whether different clones of the same cell line vary in their response to chemical insult by comparing two clones of HepG2 cells (Fig. 6). The ECACC clone is a commercially available HepG2 clone that is used by the MIP-DILI consortium for all HepG2-related studies for consistency. This allows direct comparison of results between studies conducted within the consortium. The results indicated differences between the two clones for only four of the compounds.

**Fig. 7:**
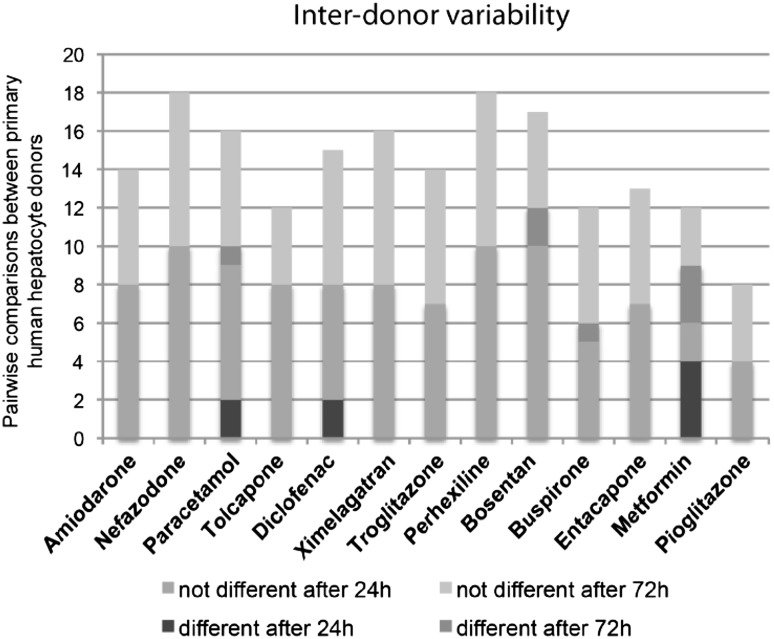
Inter-donor variability in response to the compounds is negligible. *Stacked column plot* visualizing the differences in responses of cryopreserved PHH isolated from five different donors. In total, differences are only detected in 8.1 % of pairwise comparisons suggesting that responses to chemical insult by the compounds are only marginally affected by the genetic background of the donor

**Fig. 8:**
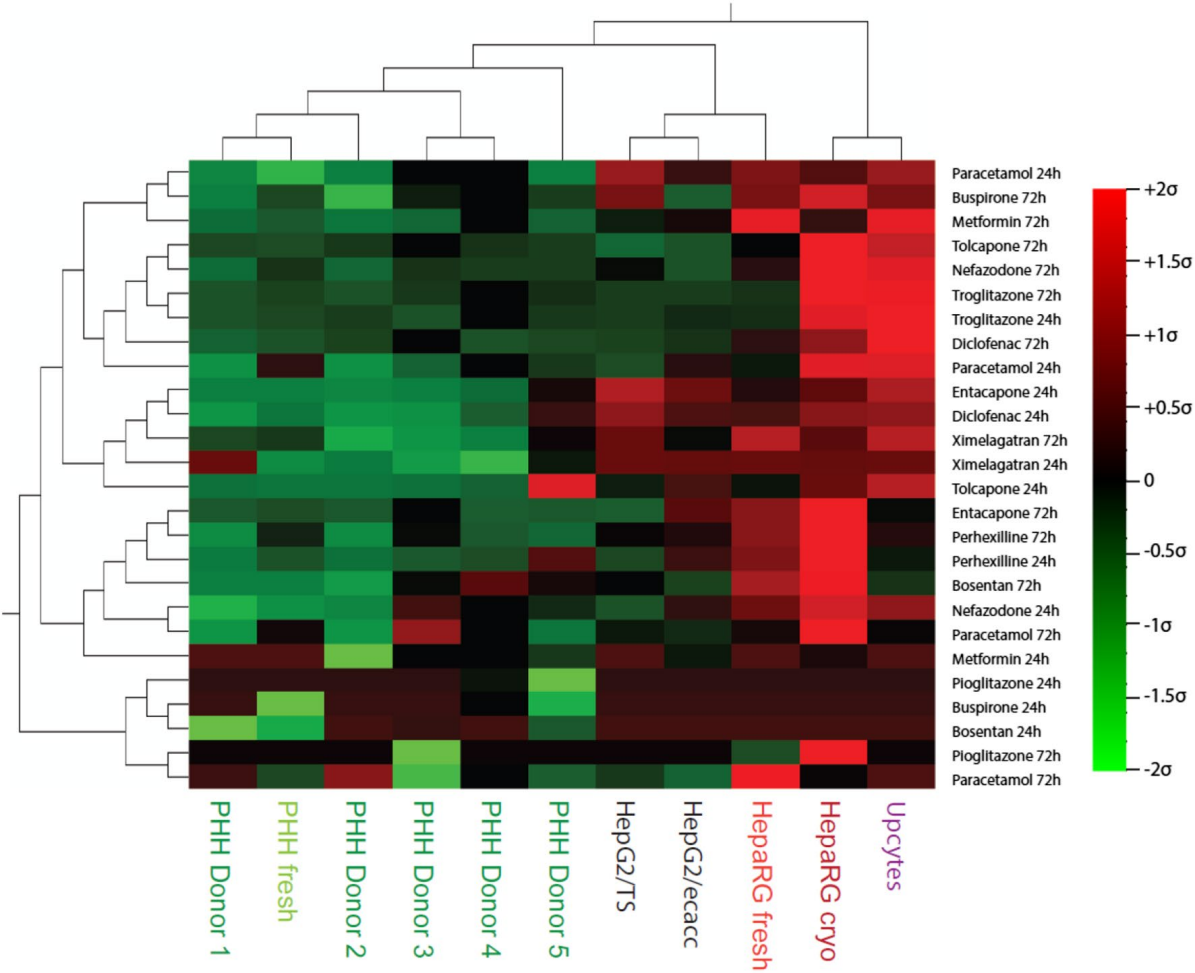
Hierarchical clustering separates primay human hepatocytes from the assessed cell line culture systems. Cell types were clustered using maximum distance measure based on their mean-centered sigma-normalized EC_50_ values as obtained from ATP measurements. Note that while the different primary human hepatocyte donors cluster closely together, HepG2s, HepaRGs, and Upcyte cells constitute a second cluster. *Coloring* indicates deviation from mean

Five cryopreserved hepatocyte donors were also assessed for inter-donor variability, and the influence of inter-donor variation was found to be only minor (8.1 %, Fig. 7). Interestingly, hierarchical clustering of the four cell types based on the EC_50_ values derived from the ATP measurements demonstrated close clustering between all the primary human hepatocyte donors while the HepG2, HepaRG and Upcyte cells constitute a second cluster (Fig. 8). This analysis further demonstrates the minor influence of donor variation between the five donors used for cryopreserved PHH in this study, as well as cryopreserved versus fresh cells (HepaRG and PHH) and clonal differences (HepG2).

## Discussion

The prediction of drug-induced liver injury (DILI) remains a challenge for medical professionals, the pharmaceutical industry, and regulatory authorities (Chen et al. 2014; Marino et al. 2001). We therefore conducted a comprehensive and unbiased acute cytotoxicity assessment of four cell models (PHH, HepG2, HepaRG and Upcyte cells, in conjunction with basic measures of cell health, namely ATP and resorufin) that are currently used in industry as part of a testing strategy to determine DILI risk. Furthermore, we used a multinational multicenter approach, involving six industry partners and one academic partner, with the majority of the cell models tested in two or more independent sites (Table 2). To our knowledge, this is the first time such a study has been done for the types of basic cell models that are commonly used in hepatotoxicity studies. Cytotoxicity profiles were generated in response to nine human DILI-implicated compounds, which were chosen according to their proposed mechanisms of hepatotoxicity in human liver, and four compounds without known human DILI liability (Table 4). Furthermore, other factors relevant in safety assessment including clonal differences between cell lines, the use of cryopreserved and fresh cells, as well as inter-donor variation, were also addressed.

We found that based on the EC_50_ values obtained from this study, none of the four cell models assessed could distinguish between DILI and non-DILI compounds. This is important as it clearly demonstrates, particularly when simple endpoints are used—a common approach during drug screening—that for the medicinal chemist, none of these cell models is suitable for the purpose of predicting the likely risk of an NCE to cause DILI in man. Thus, current in vitro models must be further developed with respect to cell composition as well as endpoints measured in order to increase predictability for the various forms of DILI that can occur in man. For the simple cell types described here, which still retain some significant advantages, e.g., cost, availability, and ease-of-use, their utility in safety assessment may be specialized to specific forms of cellular stress, e.g., mitochondrial damage, functional changes in endoplasmic reticulum, nuclear damage, and lysosomal perturbation, many of which are currently used in high-content screens, combined with appropriate endpoints. We have recently determined the basal hepatic proteome of each of the cell types assessed here, data which could be valuable in deciding how these cells are used appropriately (Sison-Young et al. 2015). For the current study, this proteomic analysis is particularly useful when interpreting the cytotoxicity data as it gives an indication of each cell type’s hepatic phenotype at the protein level which could be indicative of their functional capability. For example, we have demonstrated varying protein levels of Phase I and Phase II enzymes in each cell type which is an important consideration for the current study as several of the compounds tested are known to be hepatotoxic through the generation of reactive intermediates (Gustafsson et al. 2014; McGill et al. 2012; Tang et al. 1999; Zahno et al. 2011). However, to fully characterize each of these cell types, functional measurements would have to be performed as one cannot assume that the level of abundance of a protein directly correlates with its activity. Nevertheless, once we try to go beyond modeling the initial chemical insult in the hepatocyte and take account of DILI mechanisms including the action of non-parenchymal cells as well as blood cells, it is anticipated that either the complexity of in vitro models will have to be increased in order to gain higher sensitivity for DILI prediction or more relevant readouts will be needed in order to capture the early molecular events and pathways within the hepatocyte which eventually lead to DILI as it occurs in a patient. This is particularly important for compounds such as ximelagatran, in which inflammation and the immune system are known to play an important role (Edling et al. 2008), and our results clearly show that the cell models tested in the current study are inadequate for modeling this.

We acknowledge the importance of pharmacokinetic data in the development of better models and assessment strategies given the central role of the liver in drug disposition (Smith et al. 2005). As such, C_max_ values were factored into our analysis, thereby deriving some human exposure relevance. Based on this, we found that PHH provided the most accurate cell model in distinguishing compounds implicated in DILI, reaching detection rates of 89 % after 72 h (Fig. 3). It is worth pointing out that while the medicinal chemist would not generally have C_max_ data at an early stage in drug development, a predicted therapeutic exposure can be calculated early on in development, and theoretically this could be used as a prediction. In the absence of known pharmacological C_max_ and AUC data, basic cell models, as described here, are limited to simple and rapid early discovery screens for the ranking of chemical series within projects. Therefore, no distinction can be made between these cell models regarding sensitivity, selectivity, or relevant EC_50_/C_max_ values to predict liver safety, without a priori knowledge of the human pharmacokinetic characteristics of the chemical entities per se. We have illustrated this by the use of an unbiased panel of DILI and non-DILI reference compounds commonly used in industry, and the subsequent analysis of cytotoxicity readouts both with and without clinical pharmacokinetic data.

The importance of chronic exposure, which is more representative of the clinical setting, was clearly evident in the current study, as a clear segregation between DILI and non-DILI compounds in PHH was only observed after a 72-h exposure to the training compounds, a pattern that was not observed at the 24-h time point. This has also been demonstrated by Holmgren and colleagues (Holmgren et al. 2014) in human stem cell-derived hepatocyte-like cells (HLCs) wherein a shift in the cytotoxicity curves of the HLCs after a 7 or 14 day exposure to drugs was observed compared with HLCs as well as HepG2 cells that have been exposed to the same drugs for 48 h. It would therefore be worthwhile in future studies to perform a chronic study in order to assess whether each of the cell models tested here can better distinguish between DILI and non-DILI compounds under chronic rather than acute exposure. In addition to the cell models tested in the present study, HLCs are also under assessment for their potential as an in vitro tool for predicting DILI risk by several groups in the field (Pradip et al. 2016; Szkolnicka et al. 2014).

Another aim of this study was to ensure that the data set generated was sufficiently robust for use as a reference tool for the evaluation of initial markers of chemical insult in simple cell models, and to benchmark more complex models of DILI. Importantly, we detected different degrees of inter-laboratory variation across the cell types assessed (Fig. 4). The largest inter-laboratory variation was observed in the PHH (48.5 %), followed by the HepG2/ECACC cells (23.4 %). It is not unexpected that the most complex and sophisticated cell in our study, the hepatocyte, shows the greatest inter-laboratory variation among the different cell models, despite the use of common reagents, plasticware and protocols. However, the variation in the HepG2 data was surprising particularly as these cells also demonstrated the highest level of intra-laboratory variation (11.54 %) among all the cell types assessed. Although identical harmonized protocols for each cell model were used by all test sites, our results show differences between laboratories that require even more stringent protocols. With the HepG2 cells, our intra-laboratory data suggests an inherent level of variability even within one type of clone. Alternatively, it is conceivable that early markers of the initial chemical insult would be less variable compared with basic cell health endpoints measured over several days in culture, and would be more appropriate (Xu et al. 2004). However, despite these variations observed and although none of the cell models could faithfully predict DILI risk, the PHH and HepG2 cells were still relatively accurate in discriminating DILI risk when exposure levels are taken into account and that this accuracy was not diminished by inter- and intra-laboratory variation.

We also assessed differences between fresh and cryopreserved HepaRG cells as well as the effect of clonal variation in the HepG2 cell line. Interestingly, cryopreservation had only minor effects on response to chemical insult in the HepaRG cells (Fig. 5). A similar observation was made between one donor of fresh and five of cryopreserved PHH (Supplementary Fig. 2). As only one donor of fresh PHH was used for comparison, additional donors would need to be tested in order to make this observation conclusive. Nonetheless, our current finding is in agreement with the minimal effects of cryopreservation in PHH that has been previously reported by other groups (Richert et al. 2006; Smith et al. 2012). Also differences between HepG2 clones when assessed by one laboratory were found to be of less importance (16.3 %, Fig. 6) than inter-laboratory variations of the same clone (23.4 %, Fig. 4a). The TS HepG2 clone used to compare with the HepG2/ECACC clone is an established HepG2 clone at one of the participating test sites and is a cell line that is routinely used for their drug screening studies. While this is not a widescale test of all HepG2 clones that are available, this does begin to address the frequently discussed, but rarely tested, issue of inter-clonal variation in response to compound exposure.

When inter-donor variation among the PHH was examined, only 8.1 % of all pairwise comparisons was observed to be different (Fig. 7). Furthermore, all five cryopreserved PHH donors and one fresh hepatocyte donor were shown to cluster together (Fig. 8), suggesting that although donor variations were observed in the response of the hepatocytes to the compounds, these variations were much less important than differences from the other cell models.

In summary, when used with simple endpoints such as the measurement of ATP and resorufin, none of the cell models currently used in industry (HepG2, HepaRG, Upcyte and PHH) can completely distinguish between established drugs with respect to their propensity to cause DILI in man, and are therefore unlikely to be able to predict the DILI hazard and risk for NCEs. However, when in vivo exposure levels are taken into account, PHH are the most accurate cell model, identifying 8 out of 9 DILI compounds as such after 72 h, but not after 24 h of exposure. Our study revealed significant inter-laboratory variation for EC_50_ values obtained in PHH, HepG2, and Upcyte cells, but not in HepaRG cells. Inter-donor and inter-clonal differences and the effect of cryopreservation were found to be of less importance than differences between the cell models. Therefore, while PHH can provide an expedient model to aid in the indication of possible DILI risks in later stages of drug development, e.g., when in vivo exposure data is already available, none of the cell models are suitable to indicate the risk of DILI early in drug development. Hence, a multicenter assessment of more reliable cell models, i.e., simple cell types such as used here, or more complex 3D or co-culture models, in conjunction with more sophisticated endpoints of molecular initiating events that report on the chemical insult in the hepatocyte is needed.

## Electronic supplementary material

Below is the link to the electronic supplementary material.

Supplementary material 1 (PPTX 688 kb)

Supplementary material 2 (DOCX 29 kb)
