# Supplementary figures and images for: A multicenter assessment of single-cell models aligned to standard measures of cell health for prediction of acute hepatotoxicity
Source: Arch Toxicol. 2016 Jun 25;91(3):1385–400. doi: 10.1007/s00204-016-1745-4 (PMC5316403; doi:10.1007/s00204-016-1745-4)

## Slide 1
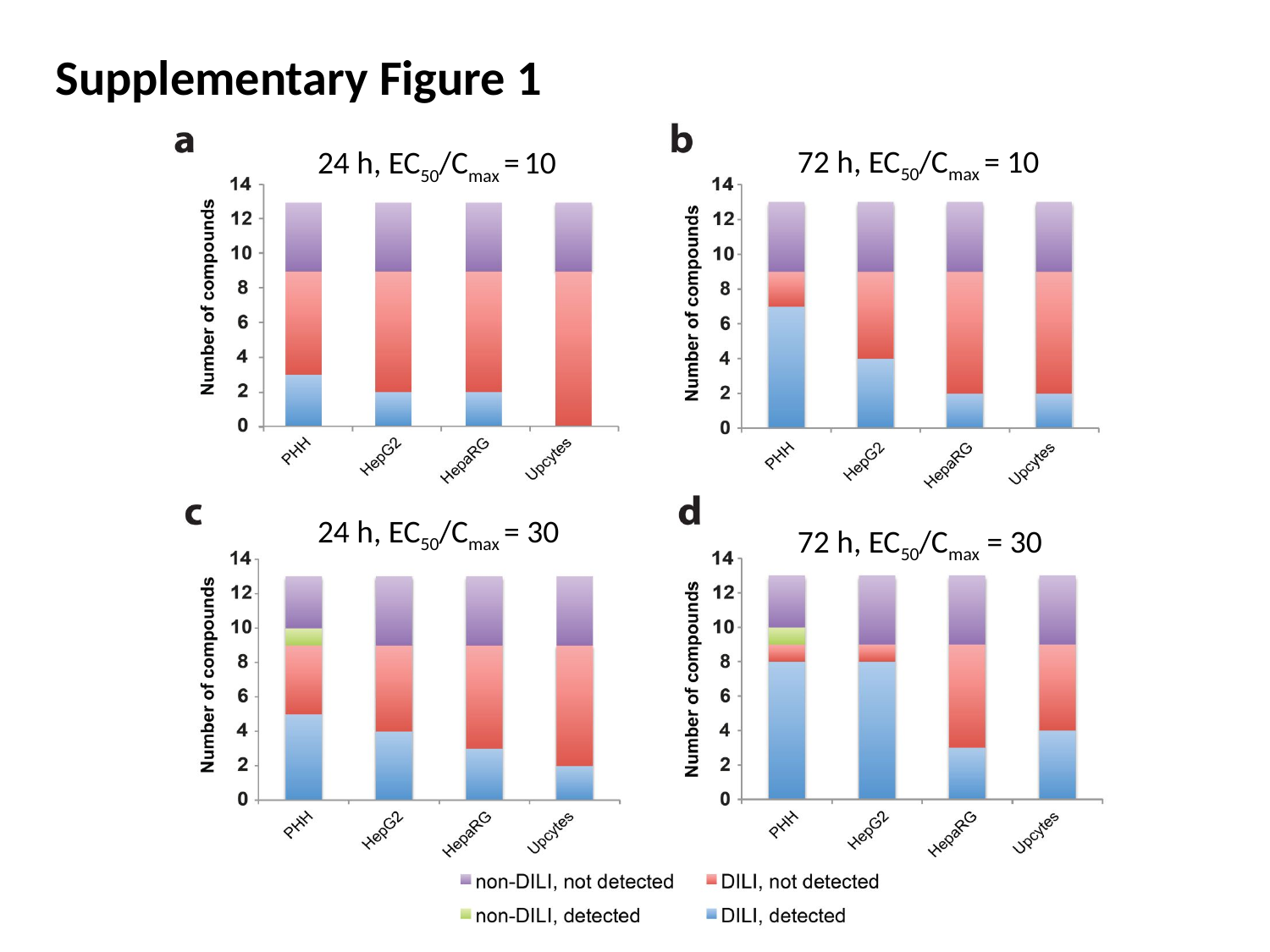

Supplementary Figure 1
72 h, EC50/Cmax = 10
24 h, EC50/Cmax = 10
24 h, EC50/Cmax = 30
72 h, EC50/Cmax = 30

## Slide 2
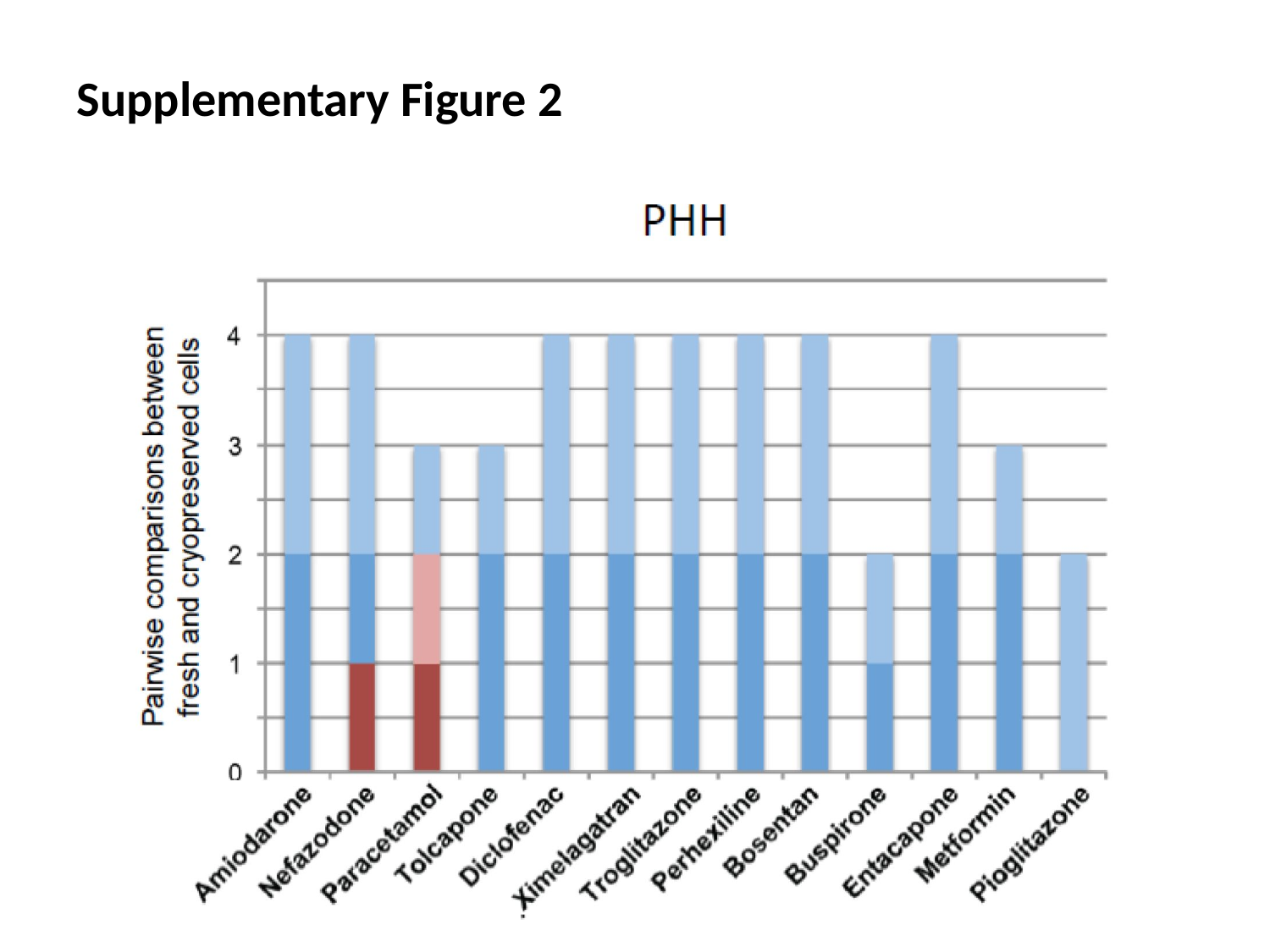

Supplementary Figure 2

Supplement: Supplementary file 1 — Supplementary material 1 (PPTX 688 kb) [file 204_2016_1745_MOESM1_ESM.pptx]
