# Supplementary material for: A multicenter assessment of single-cell models aligned to standard measures of cell health for prediction of acute hepatotoxicity
Source: Arch Toxicol. 2016 Jun 25;91(3):1385–400. doi: 10.1007/s00204-016-1745-4 (PMC5316403; doi:10.1007/s00204-016-1745-4)
Supplement: Supplementary file 2 — Supplementary material 2 (DOCX 29 kb) [file 204_2016_1745_MOESM2_ESM.docx]

**Supplementary Table 1. EC_50_ (µM) values from primary human hepatocytes, HepG2 cells, HepaRG and Upcyte cells in response to the training compounds.** Primary human hepatocytes (fresh or cryopreserved), HepG2 (ECACC or TS clone), HepaRG (fresh or cryopreserved) and Upcyte cells were exposed to each of the thirteen training compounds using a range of doses detailed in Table 4 for either 24 or 72 h. Cell viability was determined by ATP (a) and resorufin (b) assays. The EC_50_ values derived from both ATP and resorufin assays indicate the primary human hepatocytes and HepG2 cells having a very similar and more sensitive response compared with the HepaRG and Upcyte cells.

**Supplementary Figure 1 | DILI and non-DILI compounds are better distinguished when exposure levels are taken into consideration.** Stacked column plots visualizing the number of compounds classified as toxic by the different cell models based on EC_50_/C_max_ values of 10 and 30.

**Supplementary Figure 2 | Fresh and cryopreserved cells elicit overall similar responses to the compounds.** Stacked column plots showing the responses of fresh and cryopreserved PHH. On the y-axis, the number of different (shades of red) and not different (shades of blue) pairwise comparisons between fresh and cryopreserved cells are plotted. Differences in responses are detected in 6.7 % of all pairwise comparisons.
